# Test-Treat-Track-Test-Treat Strategy for Control of Schistosomiasis in Two Low-Prevalence Villages in Northwestern Tanzania

**DOI:** 10.4269/ajtmh.22-0442

**Published:** 2023-05-09

**Authors:** Jane K. Maganga, Carl H. Campbell, Teckla Angelo, Justina Mosha, Joseph R. Mwanga, Safari M. Kinung’hi

**Affiliations:** ^1^ National Institute for Medical Research, Mwanza Center, Mwanza, Tanzania;; ^2^ Schistosomiasis Consortium for Operational Research and Evaluation, Center for Tropical and Emerging Global Diseases, University of Georgia, Athens, Georgia;; ^3^ Department of Epidemiology, Biostatistics and Behavioral Sciences, School of Public Health, Catholic University of Health and Allied Sciences, Mwanza, Tanzania

## Abstract

Mass drug administration of praziquantel becomes a less attractive strategy for elimination of schistosomiasis in low-prevalence areas due to cost implications and low treatment compliance. We aimed to determine the feasibility of a Test-Treat-Track-Test-Treat (5T) strategy in two low-prevalence villages; the 5T strategy has been successfully implemented in diseases such as malaria. A total of 200 school children aged 6–12 years were randomly selected from two schools and tested for *Schistosoma mansoni* infection using the point-of-care circulating cathodic antigen test. *Schistosoma mansoni*–positive children, referred to as first-generation cases (FGCs), were tracked and treated including up to five members of their families. Second-generation cases, identified by the FGCs as their close, non-relative contacts, were also tracked, tested, and treated, including up to five members of their families. The prevalence of schistosomiasis among screened FGCs was 16.5% (33/200) in both villages. Twenty-four FGCs were included in the study. Prevalence among 94 contacts of FGCs was 46.8% (44/94). The proportion was higher in Muda than Bulunga village (61.2% versus 31.1%, χ^2^ = 10.6611, *P* = 0.005). Prevalence among SGCs and their contacts was 37.5% (9/24) and 47.1% (49/104), respectively. Overall, the 5T strategy identified 102 additional cases out of 222 tracked from FGCs, 95% of whom were treated, at a total time of 52 hours. Our data demonstrate the potential of the 5T strategy in identifying and treating additional cases in the community and hence its practicality in schistosomiasis control in low-prevalence settings at relatively low time and resources investment.

## INTRODUCTION

Schistosomiasis is estimated to be the third most debilitating tropical disease after malaria and soil-transmitted helminthiases, affecting over 250 million people globally in about 76 endemic countries.^1^^–^^7^ More than 90% of these cases occur in Africa.^1^^,^^8^^,^^9^ Tanzania ranks as the second most burdened country after Nigeria, with a recorded overall prevalence over 50%.^1^^,^^10^^,^^11^ A more recent 2022 report states that the regional prevalence across the country ranges from 12.7% to 87.6%.^12^ Based on this high prevalence, the mainstay of treatment in highly endemic regions in Tanzania from 2004^12^ has been annual mass drug administration (MDA) of praziquantel targeting primary school–going children, who carry the highest burden of infection, as per the WHO’s treatment guidelines.^13^^–^^15^ In 2006, the WHO expanded its target population to include adults in highest-risk areas in which the prevalence in school children was greater than 50%.^16^ The Lake Victoria region of Tanzania was particularly targeted with MDA programs because of recorded prevalence of *Schistosoma mansoni* above 90%.^1^^,^^17^^–^^21^ Furthermore, in 2022 the WHO, in an ambitious goal of eliminating schistosomiasis as a public health problem, published new guidelines, including a recommendation to expand preventive chemotherapy eligibility to all age groups 2 years and older.^22^

Multiple treatment initiatives have supported annual MDA for school-age children in many countries. The Schistosomiasis Consortium for Operational Research and Evaluation (SCORE) project, along with research institutes and health ministries, conducted a 5-year longitudinal study in 375 villages near Lake Victoria in Kenya and Tanzania from 2011 to 2016. These studies demonstrated that annual school-based treatment and community-wide treatment MDA effectively reduced the prevalence and intensity of *S. mansoni* infection in the study sites.^23^^–^^25^ As these areas transition from high to low prevalence, annual MDA is becoming less cost-effective and less acceptable to community members. Ideally, selective and enhanced treatment strategies, such as those recommended by the WHO for lower prevalence areas,^16^ could maintain and perhaps further lower infection levels. For example, incorporation of newer diagnostic tools in addition to microscopy-based Kato Katz (KK) stool assays would allow more sensitive detection of low-intensity infections.^26^

As an analogous comparison, malaria control efforts have greatly reduced the burden of the disease, even in areas with high transmission in Africa. These efforts have led to the paradigm shift from presumptive treatment based merely on clinical suspicion to case confirmation with a diagnostic test prior to treatment.^27^ Goals included not only to reduce drug wastage but also to prevent under-treatment of other illnesses presenting with fever. The newly introduced WHO Global Malaria Program’s initiative “Test Treat Track” (T3) is a venture to improve diagnosis, focus treatment, and strengthen disease surveillance, moving toward elimination.^28^ We postulate that this same strategy could be developed, implemented, and evaluated for implementation in areas of low prevalence for schistosomiasis. Such a strategy could be complimentary to the current WHO recommendation of providing selective treatment in low-prevalence schistosomiasis areas and would also improve surveillance of the disease.

We therefore implemented a study to determine the feasibility of conducting a Test-Treat-Track-Test-Treat (5T) strategy in two low-prevalence villages in northern Tanzania. This strategy utilizes testing for suspected cases, providing praziquantel treatment of confirmed cases, and disease tracking among contacts of confirmed cases to create and maintain an accurate surveillance system and to curtail transmission in small hotspots within the overall low-prevalence community. We used the point-of-care circulating cathodic antigen (POC-CCA) assay alongside the standard KK technique for diagnosis of *S. mansoni* infection. We hypothesized that this strategy would identify cases at a higher prevalence than the overall prevalence that would be identified through random testing in these low-prevalence villages. We further anticipate that insight gained through this pilot 5T strategy can guide the development of a protocol to strengthen the three pillars of testing, treatment, and surveillance for schistosomiasis that will be useful in areas of low schistosomiasis prevalence.

## MATERIALS AND METHODS

### Study site and subjects.

**Figure 1.**
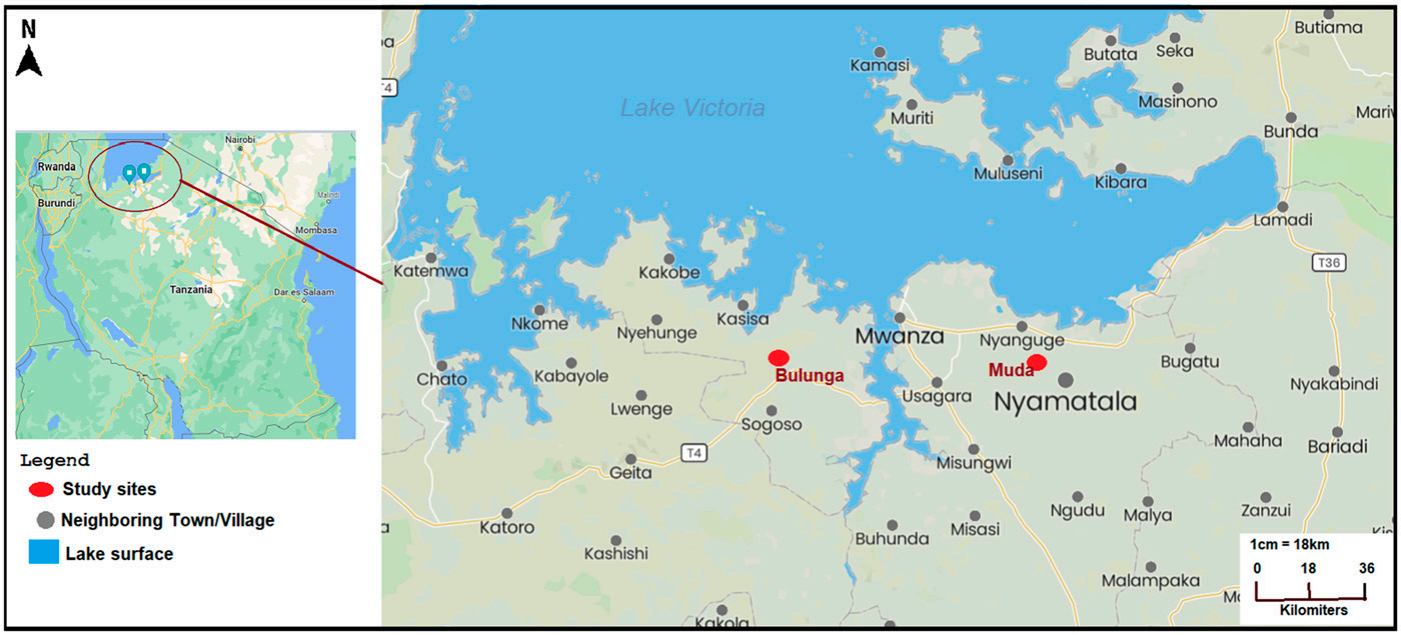
Map showing study sites and Muda and Bulunga primary schools in corresponding villages in northern Tanzania. The villages are found in Sengerema and Magu districts along the shores of Lake Victoria.

The 5T study was conducted in two low-prevalence schools and corresponding villages, Muda and Bulunga, in Magu and Sengerema districts, respectively (Figure 1). The two districts are in northwestern Tanzania along the shores of Lake Victoria. The villages were randomly selected from a list of nine low-prevalence villages based on the results of the final survey of the 5-year cross-sectional SCORE SM2 studies in Tanzania conducted from 2011 to 2016 in 150 villages. School children in these villages had an *S. mansoni* prevalence of less than 10% by the KK method. Additionally, they had a schistosome prevalence of less than 25% by urine POC-CCA assay at the initial screening for the current study. This percent cut-off for the CCA is based upon KK and POC-CCA assay comparative results in prior SCORE studies and ongoing modeling work.^29^ It is also in line with the cut-offs for low-prevalence settings in the new WHO guidelines.^22^

### Study procedure.

To verify the low prevalence of *S. mansoni* infection in the two selected schools, urine samples were first collected from 30 randomly selected children aged 6–12 years from class 1–7 in each of the schools and tested using the POC-CCA rapid test. The prevalence in both schools from these samples was confirmed to be less than 25%, as anticipated. We then collected urine from 100 randomly selected children of the same age group and from the same classes in each school (the 30 initial children included) for testing by the urine POC-CCA assay. Testing was done at school. All children who tested positive by this test in both villages were identified and considered for enrollment in the study. This number included all 10 trace-positive children in Muda. In Bulunga, which had more children who tested trace positive, we randomly selected 10 children from among them to include in the study. These enrolled children were referred to as first-generation cases (FGCs). In total 24 FGCs were included.

On the following day, each of the two study teams, comprised of a research assistant, a laboratory technician, and a community health worker (CHW), selected one FGC from the list at the school and walked home with the child to conduct contact tracing. On arrival to the child’s home, the field assistant reported the child’s positive results to the child and family and sought consent for the child to receive praziquantel treatment. Additionally, a pre-tested questionnaire on water contact activities was administered to each FGS. Global positioning system (GPS) coordinates of the FGC’s household were collected.

Given the positive results of the FGC, the field assistant used the opportunity to encourage up to five members of the FGC’s family to test for schistosomiasis. An appointment was set for the next day for the team to meet the family for testing and treatment. All people who resided in the household aged 6 years and above were eligible. Children below 6 years were excluded because of a lack of an appropriate pediatric praziquantel formulation for treating this group.^30^ In households with five people or fewer, all were given an opportunity to participate if they consented. In those with more than five people, a random selection procedure was used to get participants. Oral informed consent for testing and treatment was obtained from the contacts. Those who were found to be positive for *S. mansoni* by the POC-CCA test were offered praziquantel treatment.

After completing activities at the FGC’s home, the FGC was asked to mention at least one close friend, preferably from the same community (not necessarily attending the same primary school) with whom they shared water exposure experiences frequently. The CHW who resides in the same community and is familiar with most families in the village helped the team to locate the home of the friend mentioned by the FGC. The team visited the home to set an appointment to come and explain about the study and obtain consent for POC-CCA testing of the mentioned child and treatment of those who were found to be schistosome infected. These children were referred to as second-generation cases (SGCs).

**Figure 2.**
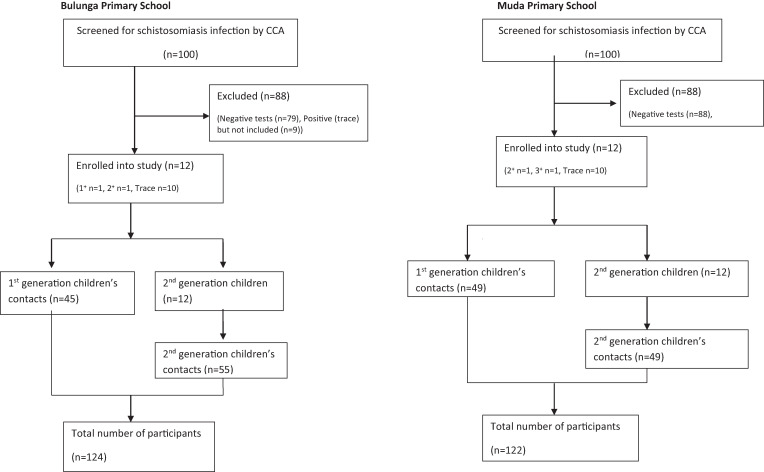
Recruitment flow chart. One hundred school children aged 6–12 years were screened in each of the two schools using the point-of-care circulating cathodic antigen (CCA) test. All children with a positive result were included in the study. In addition, 10 trace positive children were included from each of the two schools.

On the agreed day of appointment, up to five family members of the SGCs who consented were also tested by POC-CCA assay; those found positive were offered praziquantel treatment. Random selection was used to get participants in households that had more than five people. All procedures took place at the SGC’s home. GPS coordinates were also collected from the homes of the SGCs. A flow chart summarizing the recruitment procedures is summarized in Figure 2.

The time taken for the various steps from the identification and locating the FGCs and SGCs was recorded. Time was measured in terms of minutes elapsed when actual activities took place. For the FGCs, time was measured from when they were identified at the school and tracked to their homes by the research team for consent of treatment and performance of the interview.

For the SGCs, time was measured when the team left the home of the FGC and traveled to the home of the SGC to book an appointment to come and conduct the consent, testing, and treatment of the SGC. The second timepoint was the time spent to obtaining consent, testing, and treatment of the SGCs.

Participants who were found positive from the POC-CCA test and who accepted treatment were given a directly observed single dose of praziquantel at 40 mg/kg body weight.

### Field and laboratory procedures.

One urine sample was collected from each participant and tested for *S. mansoni* infection using the POC-CCA test (Rapid Medical Diagnostics, Pretoria, South Africa) at school and participant households. Two drops of urine were added to the well of the testing cassette, and the test was read at 20 minutes. Tests were considered invalid when the control band did not appear. A result was considered negative if a control band was seen in the absence of a test band. For positive results, the intensity of the test band was compared with that of the control band. A score of “trace” was given when the test band was barely visible, 1+ when the test band was less intense than the control band, 2+ when the test band was of equal intensity as the control band, and 3+ when the test band was more intense than the control band. Based upon the manufacturer’s instructions and the practice of other investigators working in similar prevalence settings, we based our conclusions on the results of the POC-CCA and considered “trace” results to be positive. Our goal was to detect uncured or re-infected persons living in communities that had implemented multiple rounds of community-wide MDA.^31^^–^^33^

In parallel, for the FGCs one stool sample was collected from each participant and examined for *S. mansoni* infection using the KK technique.^34^ Each child provided one stool sample, from which four slides were prepared. Smears were placed on glass slides using a template delivering 41.7 mg of the fecal matter. These were then examined using a microscope for *S. mansoni* eggs by a trained parasitologist at the National Institute for Medical Research in Mwanza.

For *Schistosoma haematobium* infection diagnosis, 10 mL of urine were processed using the urine filtration method. The slides with the filters were then examined microscopically for *S. haematobium* eggs by a trained parasitologist. The KK and urine filtration methods were only used in FGCs for diagnostic comparison purposes with the POC-CCA.

### Statistical analyses.

Data were recorded on paper and then entered into the Census and Survey Processing System software (CSPro, U.S. Census Bureau, Suitland, MD). All data were double entered. Data were then cleaned and analyzed using Stata/IC version 21 (Stata, College Station, TX). Frequencies of categorical variables and the median and interquartile ranges for continuous variables were quantified using descriptive statistics. Pearson χ^2^ tests were used to determine the association between schistosomiasis and demographic variables. Significance of tests was considered at *P* < 0.05.

## RESULTS

### Prevalence of *S. mansoni* among children screened at the primary schools (FGCs).

Of the 200 children screened for *S. mansoni* in the two schools, 33 (16.5%) were positive by the POC-CCA method when trace results were also considered positive, and four were positive (2%) when trace results were considered negative. In comparison, only one (0.5%) FGC was positive by the KK method; this case was from Muda primary school (Table 1). This child with positive stool egg microscopy results was also POC-CCA positive. Only two (1%) of the participants were *S. haematobium* egg positive; both were from Bulunga primary school. One of these two also had a positive POC-CCA result, implying co-infection with both schistosome species. All FGCs received praziquantel treatment.

**Table 1:** Prevalence of schistosomiasis by method of testing among first-generation cases

Method	Bulunga (*N* = 100)	Muda (*N* = 100)	Total (*N* = 200)
CCA (with trace as positive), *n* (%)	21 (21)	12 (12)	33 (16.5)
CCA (with trace as negative), *n* (%)	2 (2)	2 (2)	4 (2)
Kato Katz (*Schistosoma mansoni* ova), *n* (%)	0	1 (1)	1 (0.5)
Urine filtration (*Schistosoma haematobium* ova), *n* (%)	2 (2)	0	2 (1)

CCA = circulating cathodic antigen.

### Socio-demographic characteristics of FGCs.

Among the 33 eligible children identified from screening 200 children, 12 *S. mansoni*–positive children from each of the two schools were enrolled in the study (Table 2). Fourteen (58.3%) were female, and the median age was 11 (9.5–12) years. Overall, most children (54.2%) reported to have lived in their respective villages for longer than 10 years. A shallow uncovered well was the water source commonly reported by the children (91.7%), followed by the river (54.2%). All children reported that they usually step into water of the water sources they visit without wearing any protective gear on their feet. All children reported that they had a toilet in their households. The distributions of other socio-demographic characteristics are shown in Table 2.

**Table 2:** Socio-demographic characteristics of first-generation cases

Participant characteristics	Bulunga school (*N* = 12)	Muda school (*N* = 12)	Total (*N* = 24)
Sex, *n* (%)			
Male	7 (58.3)	3 (25)	10 (41.7)
Female	5 (41.7)	9 (75)	14 (58.3)
Age in years, median (IQR)	10.5 (9–12)	11 (9.5–12)	11 (9.5–12)
Length of stay in village, *n* (%)			
0–2 years	1 (8.3)	0 (0)	1 (4.2)
3–5 years	2 (16.7)	0 (0)	2 (8.3)
6–10 years	3 (25.0)	5 (41.7)	8 (33.3)
> 10 years	6 (50.0)	7 (58.3)	13 (54.2)
Main source of water, *n* (%)			
Tap water	0	8 (66.7)	8 (33.3)
Lake	0	0	0
Deep well, covered	0	1 (8.3)	1 (4.2)
Shallow well, uncovered	12 (100.0)	10 (83.3)	22 (91.7)
River	8 (66.7)	5 (41.7)	13 (54.2)
Type of toilet in household, *n* (%)			
Modern water flushing toilet	4 (33.3)	7 (58.3)	11 (45.8)
Pit latrine	8 (66.7)	5 (41.6)	13 (39.4)
No toilet	0	0	0
Usually step in water, *n* (%)			
Yes	12 (100.0)	12 (100.0)	24 (100.0)
No	0	0	0
Wear protective gear			
Yes	0	0	0
No	12 (100.0)	12 (100.0)	24 (100.0)

### Prevalence of *S. mansoni* among contacts of FGCs.

A total of 94 contacts were tracked directly from FGCs in both villages. The overall prevalence of schistosomiasis among these contacts of FGCs was 46.8% (44/94). There were more positive contact cases found in Muda village (61.2% of contacts) than in Bulunga village (31.1%, χ^2^ = 10.6611, *P* = 0.005) (Table 3). The proportion of positives was higher in females than in males, although this difference was not statistically significant (χ^2^ = 1.8652, *P* = 0.172). Among the FGCs’ contacts who were found positive, 3 (6.8%) refused to be treated.

**Table 3:** Schistosomiasis test results and characteristics of first-generation cases’ household contacts, second-generation cases, and their contacts

Group	Age in years, median (IQR)	*P* value	Sex, *n* (%)			Village, *n* (%)		
			Male	Female	*P* value	Bulunga	Muda	*P* value
FGCs’ household contacts who tested positive	15.5 (9.5–36.5)	0.7300	15 (38.5)	29 (52.7)	0.172	14 (31.1)	30 (61.2)	0.005
FGCs’ household contacts who tested negative	17 (9–39)		24 (61.5)	26 (47.3)		31 (68.9)	19 (38.8)	
SGCs who tested positive	12 (11–12)	0.4292	4 (36.4)	5 (38.5)	0.916	2 (16.7)	7 (58.3)	0.035
SGCs who tested negative	11 (9–13)		7 (63.6)	8 (61.5)		10 (83.3)	5 (41.7)	
SGCs’ household contacts who tested positive	13 (8–33)	0.5082	21 (42.9)	28 (50.9)	0.412	19 (34.5)	30 (61.2)	0.007
SGCs’ household contacts who tested negative	15 (10—31)		28 (57.1)	27 (49.1)		36 (65.5)	19 (38.8)	

FGC = first-generation case; SGC = second-generation case.

### Prevalence of *S. mansoni* among SGCs and their contacts.

A total of 24 SGCs were tested in both villages. The prevalence of schistosomiasis among these children was 37.5% (9/24). There were more positive cases in Muda village (χ^2^ = 4.4444, *P* = 0.035) and among females; however, the sex difference was not statistically significant (χ^2^ = 0.0112, *P* = 0.916).

There was a total of 104 SGCs contacts cases from both schools. Of these, 49 (47.1%) had schistosomiasis. Muda village had more positive cases than Bulunga (61.2% versus 34.5%, χ^2^ = 7.4024, *P* = 0.007).

All of the SGCs who were positive accepted treatment; however, two (4.1%) of their positive contacts refused treatment. Reasons for refusal included difficulty of swallowing drugs because they were big; unpleasant side effects including dizziness, vomiting, and weariness; and inability of drugs to protect from re-infection.

Overall, the number of positive cases found by tracking FGCs was 102 out of 210 (48.6%) (Table 3).

### Time to locate the children.

**Figure 3.**
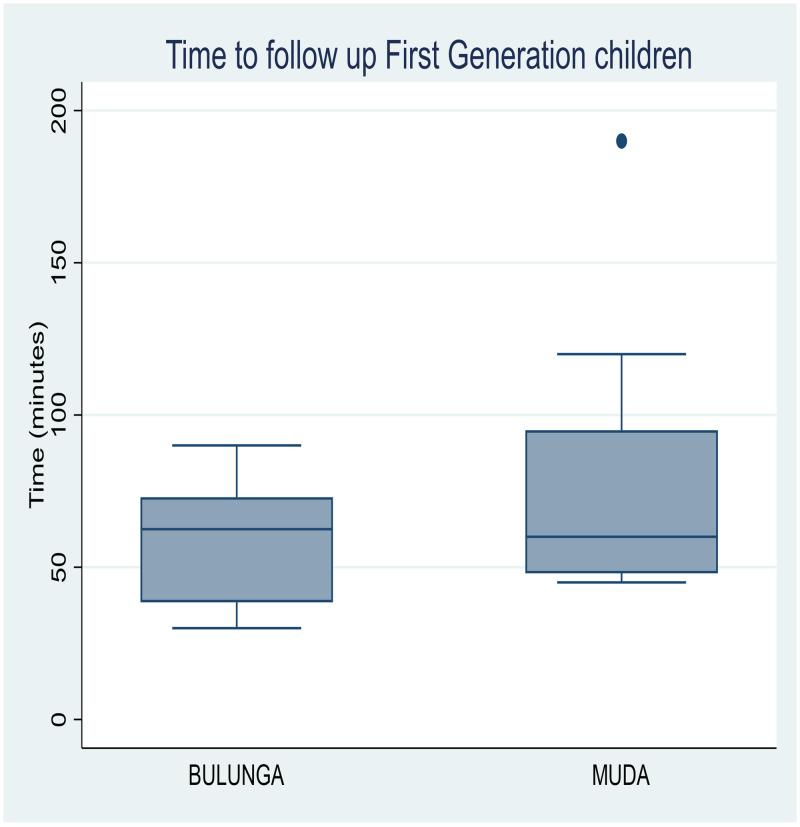
Time to follow up, first-generation cases, including time to identify the children at their primary schools, to conduct an interview, and to follow them up to their homes for treatment. The box and whisker plots show median time for this process was 62.5 minutes in Bulunga and 60 minutes in Muda.

**Figure 4.**
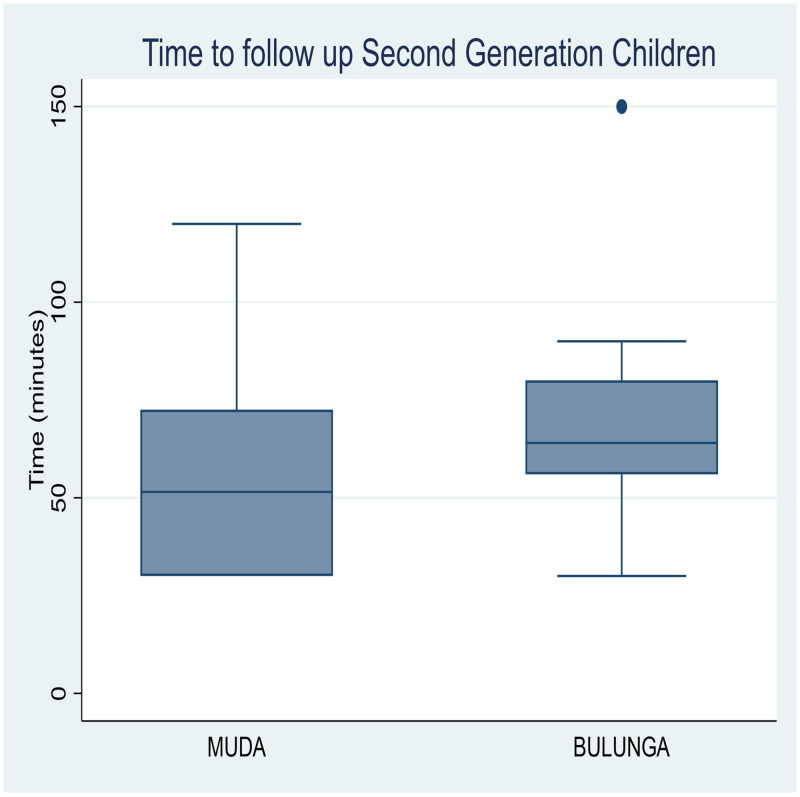
Time to follow-up, second-generation cases. The median time to follow up second generation children from identification to testing and treatment was 80 minutes at Bulunga and 58 minutes at Muda, as shown in the box and whisker plots.

The median time taken to identify FGCs at their primary schools, to conduct an interview, and to follow them up to their homes to conduct the interview and treatment was 62.5 minutes in Bulunga and 60 minutes in Muda. Identifying SGCs took a median of 80 minutes at Bulunga and 58 minutes at Muda (Figures 3 and 4). Therefore, to test, track, and treat all 48 FGCs and SGCs required a total time of 52 hours, which led to treatment of 97 additional cases of schistosomiasis, including the SGCs.

## DISCUSSION

Using the novel 5T strategy, we were able to identify 102 additional cases in the community that were associated with FGCs. Tracking infected children’s contacts produced a high-risk population that, even in these low-endemic villages, had an overall high prevalence (48.6%). These people, if left untreated, could be a source of continued community transmission, bringing the community back to pre-intervention prevalence levels within a short time period following treatment. Furthermore, it is likely that most asymptomatic cases will still experience some form of morbidity and disability due to the chronic nature of the disease, so that treating these individuals can be expected to benefit their health as well as the overall community.^35^

The findings of this study also suggest that the 5T strategy is feasible and cost-effective. The time taken to track all the FGCs and SGCs to their homes and perform the various procedures outlined above was equivalent to about one-third of 1 month effort for one person working 8 hours a day. We are aware of one other study that used the 5T strategy for schistosomiasis control in Egypt.^36^ In contrast to our findings, this study found the process of tracking and tracing children to be labor intensive in terms of the person-time requirements. However, similarly, the study in Egypt concluded that the 5T strategy could be feasible, achievable, and cost-effective in moving toward elimination of schistosomiasis.

The prevalence of *S. mansoni* among the FGCs was very low in the Muda and Bulunga villages by both the KK and POC-CCA methods. To a large extent, these results were expected because these villages had received multiple rounds of annual MDA of praziquantel through the work of the SCORE/NIMR project in coordination with the Ministry of Health over a 5-year study period. Nevertheless, in parallel comparison, the POC-CCA test results revealed a higher prevalence than the KK. Our results agree with findings from other studies that have shown that the KK method is less sensitive than the POC-CCA method in areas with low prevalence and light infection intensity^29^^,^^31^^,^^37^^–^^40^ and that dependence on KK may consequently underestimate the true prevalence of the disease.^41^ A five-country evaluation of schistosome diagnostics documented that the increased sensitivity of the POC-CCA over the KK was highest among schools that had prevalence between 10% and 25%.^38^ The low sensitivity of the KK can be attributed to the daily fluctuations in egg excretion, the heterogeneity of egg distribution in stool samples, and its inability to diagnose recent infections in which schistosome worms have not started producing eggs.^37^^,^^40^^,^^42^^–^^45^ Furthermore, costs of accurate diagnosis by KK in low-intensity settings would likely increase due to the need to examine multiple stool samples per person to increase sensitivity.^46^

The prevalence of *S. haematobium* among the FGCs was also very low in these two villages, at 1% by the urine filtration method. In addition, both cases were from one village, Bulunga. The findings are in keeping with what other authors have reported about areas around the shores of Lake Victoria^47^ and the known dependence of the two schistosome species on preferred habitats of their snail intermediate hosts. The *Biomphalaria* species, which are intermediate hosts for *S. mansoni*, are found along the shores of Lake Victoria, whereas the *Bulinus* species, which are intermediate host for *S. haematobium*, are commonly found inland in streams and ponds.^48^^,^^49^ Both of our study villages lie along the shores of Lake Victoria. A possible reason for *S. haematobium* infection in these two participants could be that they had recently visited a *S. haematobium*–endemic area. Unfortunately, travel history was not elicited.

Treatment uptake was satisfactory because only a small fraction (4.9%) of all positive contacts refused treatment. The reasons for refusal included too many drugs that were big and therefore difficulty to swallow; unpleasant side effects, including dizziness, weariness, and vomiting such, that they would not be able to continue with their work for the day; reliance on traditional herbs; and mistrust of the drugs because even after taking the drugs people still get sick with the disease later. These reasons for non-uptake are similar to those reported in other studies.^50^^,^^51^ Some of these reasons highlight the likelihood that participants who refused treatment may have had little understanding of schistosomiasis and its treatment. Providing community education on the subject may increase treatment uptake,^52^ further enhancing control and elimination efforts.

One limitation of our study, as reported by others, was the use of POC-CCA to quantify infection based on positive band intensity, especially when it came to decisions around faint bands (“trace results”).^32^^,^^41^^,^^46^^,^^53^ The predicament has always been whether to consider these as positive or negative results, even though the manufacturer recommends that “trace” be reported as positive. Investigators in a Brazilian population with light infections have maintained that trace results cannot be defined as positive or negative and recommend verification by lyophilization of urine before testing.^54^ However, our findings concur with many other studies whose findings corroborate that, even when trace results were considered negative, the prevalence of POC-CCA was still higher than that of the KK.^1^^,^^55^^,^^56^ Our decision to classify “trace” results as positive was further supported by the finding that the prevalence of schistosome infection was higher among the contacts of these trace-positive cases than would have been expected among other adults in low-prevalence communities. This suggests that in low-endemic settings, the POC-CCA assay may be a valuable tool for *S. mansoni* testing, both for predicting true prevalence estimates and for identifying those requiring treatment.^31^^–^^33^^,^^35^^,^^46^

In conclusion, we acknowledge that the number of children screened and those subsequently followed was small and may affect the external validity of our study results. However, our data demonstrate the feasibility of the 5T strategy in identifying positive cases in the community and reducing drug costs and waste that would be incurred by using a blanket MDA strategy in areas with low prevalence. In fact, our study may have been able to identify even more positive cases if we had not limited the number of participants to be tested to five per household due to limitation of available funds. Further, due to the relatively low time investment needed to identify and treat multiple cases in two villages (1/3 of one person-month) and the use of rapid point-of-care tests, the 5T strategy holds potential for scale-up and adoption by health ministries in areas approaching schistosomiasis elimination.

Future studies could include a more formal cost-effectiveness analysis, which would account for additional expenses, such as transport to reach villages and the cost of POC-CCA assays and other diagnostic tools. This would be a logical next step given the potential of the 5T strategy, on a wide scale, to identify and treat infected individuals in the community who are likely to remain as a source of ongoing community transmission.

## Financial Disclosure

This work received financial support from the University of Georgia Research Foundation, Inc., which was funded by the Bill and Melinda Gates Foundation for the Schistosomiasis Consortium for Operational Research and Evaluation (SCORE) (Grant Number OPP50816). J. K. M. is also supported by the Fogarty International Centre of the National Institute of Health under award number D43TW011295.
